# A telemedicine bridge clinic improves access and reduces cost for opioid use disorder care

**DOI:** 10.1016/j.dadr.2024.100227

**Published:** 2024-03-12

**Authors:** Michael J. Lynch, Dominic Vargas, Mary E. Winger, Justin Kanter, Jessica Meyers, James Schuster, Donald M. Yealy

**Affiliations:** a UPMC Health Plan, USA; b UPMC Department of Emergency Medicine, University of Pittsburgh, USA; c UPMC Center for High-Value Health Care, USA

**Keywords:** Telemedicine, Bridge clinic, Buprenorphine, Opioid use disorder

## Abstract

**Objective:**

We evaluated the impact of a telemedicine bridge clinic on treatment outcomes and cost for patients with opioid use disorder. Telemedicine bridge clinics deliver low-barrier rapid assessment of patients with opioid use disorder via audio-only and audiovisual telemedicine to facilitate induction on medication therapy and connection to ongoing care.

**Methods:**

A pre-post analysis of UPMC Health Plan member claims was performed to evaluate the impact of this intervention on the trajectory of care for patients with continuous coverage before and after bridge clinic visit(s).

**Results:**

Analysis included 150 UPMC Health Plan members evaluated at the bridge clinic between April 2020 and October 2021. At least one buprenorphine prescription was filled within 30 days by 91% of patients; median proportion of days covered by buprenorphine was 73.3%, 54.4%, and 50.6% at 30, 90, and 180 days after an initial visit compared to median of no buprenorphine claims 30 days prior among the same patients. Patients had an 18% decline in unplanned care utilization 30 days after initial Bridge Clinic visit, with a 62% reduction in unplanned care cost per member per month (PMPM), 38% reduction in medical cost PMPM, and 10% reduction in total PMPM (medical + pharmacy cost) at 180 days. Primary care, outpatient behavioral health, and laboratory costs increased while emergency department, urgent care, and inpatient costs declined.

**Conclusion:**

Utilization of a telemedicine bridge clinic was associated with buprenorphine initiation, linkage to ongoing care with retention including medication treatment, reduced unplanned care cost, and overall savings.

## Highlights

• A Telemedicine Bridge Clinic effectively engaged patients on buprenorphine therapy.
• The Bridge Clinic was associated with sustained increases in buprenorphine use.
• Patients treated by the Bridge Clinic had increased planned care utilization.
• Unplanned care use decreased significantly after Bridge Clinic intervention.
• Bridge Clinic use was associated with declining costs of care.

## 1 Introduction

Overdose deaths are rising in the US, with approximately 107,000 Americans dying in 2021. Of those, more than 80,000 deaths resulted from opioids—a ~50% increase compared to 2019 (Ahmad et al., 2023). Despite availability, most people with opioid use disorder (OUD) do not receive evidence-based and effective recovery medication treatments, including buprenorphine (National Academies of Sciences, Engineering, and Medicine, 2019, Substance Abuse and Mental Health Services Administration, 2022). Significant barriers to treatment include geography, transportation, cost, and stigma (Substance Abuse and Mental Health Services Administration, 2022, Hall et al., 2021).

Among available interventions, increasing access to medications for OUD (MOUD) is expected to have the largest impact on reducing overdose deaths followed by expanded distribution of naloxone and other harm reduction measures (Ballreich et al., 2020). Innovative care delivery models and systems of care that consider and remedy traditional delays and obstacles to OUD care are needed to optimize patient access. An increasingly viable model is the bridge clinic. Bridge clinics deliver short-term, low-barrier care to patients who might otherwise face challenges engaging and remaining in treatment. Bridge clinics are non-stigmatizing environments that offer harm reduction services, immediate access to MOUD, assessment of co-occurring physical and behavioral health conditions, and services to address social drivers of health with linkage to community resources (Jakubowski and Fox, 2020). Bridge clinics are commonly located in emergency departments or outpatient clinics affiliated with hospitals.

During the COVID-19 pandemic, temporary Drug Enforcement Administration (DEA) rules suspending in-person evaluation requirements for the initiation of controlled substance treatments, including both audio and audiovisual buprenorphine prescribing, allowed for expansion of bridge clinic services to a virtual environment (Second Temporary Extension). Buprenorphine therapy for OUD provided through a telemedicine platform can overcome some treatment obstacles, improve care engagement and retention, decrease overdose and mortality rates, and lower health care cost (Hailu et al., 2023, Guillen et al., 2021, Jones et al., 2022, Jones et al., 2023). Bridge clinics, especially those using telemedicine, can serve as a safety net to close gaps in the continuum of care which may arise for myriad reasons and cause diminished access to proven lifesaving care. Also, they can seamlessly connect patients with the larger network of existing treatment, recovery, and harm reduction services.

Published reports for two OUD telemedicine bridge clinics showed high engagement in treatment: 89% and 96% of patients filled a buprenorphine prescription within 1 month of the virtual visit; 69% and 77% of patients filled two or more prescriptions, respectively (Lowenstein et al., 2022, Lynch et al., 2022). A review of both in-person and telemedicine low threshold bridge clinics indicated these programs were acceptable to patients, improved the continuum of care by overcoming barriers to treatment, and effectively initiated MOUD (Taylor et al., 2023). The same review suggested that further investigations were needed to understand and document how bridge clinics facilitate connections to ongoing care and impact important outcomes beyond initial bridge clinic engagement including emergency department (ED) utilization, inpatient admissions, and healthcare cost.

The UPMC Department of Emergency Medicine, with support from UPMC Health Plan, created the UPMC Medical Toxicology Telemedicine Bridge Clinic (Bridge Clinic) in April 2020 to rapidly engage patients with OUD and coordinate care with local treatment providers and other stakeholders throughout Pennsylvania (Jones et al., 2022). The non-profit Bridge Clinic differs from other telemedicine programs by emphasizing immediate access to care and transition to local long-term providers, augmenting rather than substituting for local treatment. We sought to describe outcomes for patients with OUD evaluated at a telemedicine Bridge Clinic serving a diverse population in many different areas. We used health insurance claims data to evaluate medication access and healthcare cost for patients both before and after bridge clinic intervention. These observations can likely inform real world impact of a telemedicine-based OUD bridge clinic.

## 2 Methods

The study population included Pennsylvania residents who were evaluated at least once by the Bridge Clinic and were continuously enrolled in UPMC Health Plan for ≥3 months before and ≥3 months after initial Bridge Clinic visit 4/27/2020 through 10/31/2021. Members who did not have continuous UPMC Health Plan coverage during the evaluation period were excluded. We used health insurance claims to identify proportion of days covered (PDC) by buprenorphine prescription (Prieto-Merino et al., 2021) and costs of care per member per month (PMPM; cost divided by total number of individual member months in the study period). Cost outcomes at all evaluation periods included unplanned care, medical cost, and total cost (medical cost + pharmacy cost) PMPM. Medical cost was further analyzed by service category and substance use disorder (SUD)-specific claims using ICD-10 codes for opioid, alcohol, sedative-hypnotic, and stimulant use disorders. Unplanned care cost included ED and urgent care visits, medical transport, and both physical and behavioral health inpatient. We describe differences in healthcare cost before and after Bridge Clinic engagement using point estimates and 95% confidence intervals (95%CIs). Given the unique nature of the bridge clinic model, a comparison group could not be developed using claims data as an initial visit with a treatment provider would be an outcome of the bridge clinic and, therefore, would not be a comparable index event. The UPMC Quality Review Committee approved the project and publication as a quality improvement initiative.

## 3 Results

Of 173 total UPMC Health Plan members evaluated at the Bridge Clinic during the evaluation period, 150 met inclusion criteria. Members who were not enrolled for ≥3 months pre-/post-Bridge Clinic visit (n=23) were excluded. Most patients had coverage through Medicaid (78%) and had a co-occurring diagnosis of a serious persistent mental illness (61%). Demographics and buprenorphine prescription outcomes are in Table 1. Most (68%) members had only one visit while the average number of visits to the Bridge Clinic was 1.86. A Bridge Clinic visit was associated with 91% of patients filling ≥1 buprenorphine prescription within 30 days. Mean and median PDC for buprenorphine were observed to be higher at all assessment time points after Bridge Clinic evaluation compared to prior, rising from a median of 0 and mean of 20.7% in the 30 days before the Bridge Clinic to a median of 73.3% (95%CI= 66.2%, 80.0%) and mean of 65.1% (95%CI= 58.0%, 72.2%) of days with buprenorphine in the 30 days after (Table 1). While PDC declined over 180 days, it remained >50% at 180 days after an initial Bridge Clinic visit.

**Table 1:** Demographics and Proportion of Days Covered for Prescription Buprenorphine.

n=150				
Demographics		N (%), range (mean) or mean		
	Age Range	18–66 (38.15)		
	White/Caucasian	131 (87%)		
	Black/African American	18 (12%)		
	Asian	1 (1%)		
	Serious Persistent Mental Illness (SPMI)	91 (61%)		
	Medicaid	117 (78%)		
	Commercial	18 (12%)		
	Special Needs Plans (SNP)	9 (6%)		
	Community Health Choices (CHC)	4 (3%)		
	Medicare	2 (1%)		
	Appointments/member	1.86		
Proportion of Days Covered with Buprenorphine				
		Median	Mean	95% CI
	180 days pre-Bridge	1.7%	24.8%	n/a
	90 days pre-Bridge	0.0%	24.3%	n/a
	30 days pre-Bridge	0.0%	20.7%	n/a
	30 days post-Bridge	73.3%	65.1%	58.0, 72.2
	90 days post-Bridge	54.4%	53.6%	45.7, 61.5
	180 days post-Bridge	50.6%	50.8%	42.8, 58.8

**Fig. 1:**
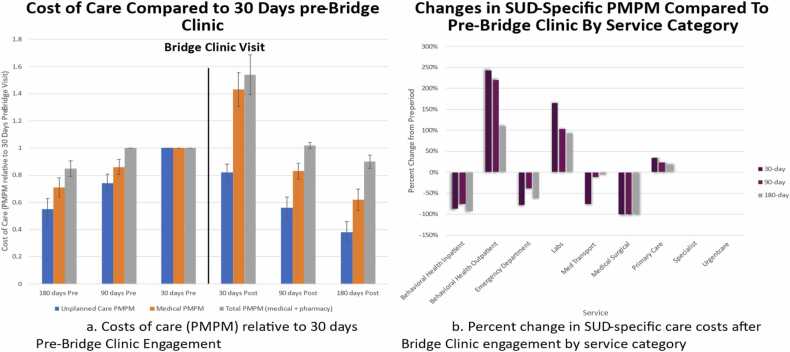
Summary of changes in care costs before and after initial Bridge Clinic engagement.

Higher medical and total cost PMPM were observed in the 30 days after the first Bridge Clinic visit, driven primarily by increased behavioral health outpatient engagement, while unplanned care cost was noted to be immediately lower (Fig. 1a). Following the initial increase, cost trends were observed to decline during the ensuing 180 days. A Bridge Clinic visit was associated with a 62% (95%CI=54.2%, 69.8%) reduction in unplanned care cost PMPM, a 38% (95%CI=30.2%, 45.8%) reduction in medical cost PMPM, and a 10% (95%CI=5.2%, 14.8%) reduction in total cost PMPM (Fig. 1a). Bridge clinic use was also associated with significant declines in medical/surgical hospitalization, behavioral health hospitalization and ED visit costs for substance use disorder (SUD)-related diagnoses, while outpatient behavioral health, primary care, and laboratory service costs increased at all assessment time points after Bridge Clinic evaluation compared to the 30 days before an initial Bridge Clinic visit (Fig. 1b).

## 4 Discussion

Virtual OUD bridge clinics are a novel telemedicine model that provide timely care when traditional care is unavailable. The UPMC Telemedicine Bridge Clinic is flexible and responsive to patient needs. Patients self-schedule by phone or a 24/7 online registration portal for an audio or audiovisual evaluation depending upon their preferences and capabilities. An addiction-trained DEA-licensed provider addresses physical health needs including withdrawal management and medication therapy. A substance use disorder assessment with referral to local care is facilitated by an outpatient care coordinator (OCC). The Bridge Clinic is led by a physician director with administrative support from UPMC Department of Emergency Medicine. Physicians and Advanced Practice Providers are scheduled hourly with time adjusted to accommodate patient demand. During the visit, patient demographics, electronic health record, and Prescription Drug Monitoring Program data are used to verify patient identity. Prescriptions for buprenorphine, naloxone, and other medical therapies are sent to the patient’s preferred pharmacy. Patients may return for additional Bridge Clinic treatment when facing delays in local care though typically not for more than 3–4 consecutive visits over the course of a month. The Bridge Clinic is supported by insurance reimbursement and grants awarded after the time-period that was reviewed.

Buprenorphine is the only prescription medication in the U.S. proven to improve withdrawal, morbidity, and mortality in patients with OUD (National Academies of Sciences, Engineering, and Medicine, 2019). Increasing access to buprenorphine is a critical intervention to reduce overdose deaths (Ballreich et al., 2020). Rapid access to medication therapy can increase the likelihood that patients will follow through with subsequent treatment. There is significant attrition when patients are forced to wait even 2 days to access MOUD (Roy et al., 2020). Prior bridge clinic evaluations showed rapid buprenorphine initiation, but impact on other OUD treatment outcomes are now better detailed in a real-world setting. The Bridge Clinic is likely to engage patients who otherwise would have been lost to care while awaiting in-person treatment. In addition to delivering initial low-barrier care with evidence-based medication therapy, a primary goal of the Bridge Clinic is to connect patients with a treatment program that meets their ongoing needs. Sources of Bridge Clinic referrals included drug treatment programs, county-based drug and alcohol commissions, jails, harm reduction programs, EDs, and word of mouth from patients who had used the program. Some patients were experiencing a gap in existing care while others were seeking to initiate treatment. Due to its flexibility and rapid low threshold accessibility, the Bridge Clinic has served as a safety net for Pennsylvanians seeking to initiate or continue MOUD care.

Claims analysis showed increased outpatient behavioral health and primary care services cost with reductions in ED and hospital cost while maintaining access to MOUD following Bridge Clinic visit. These findings are consistent with engagement and retention in recommended treatment. Bridge Clinic intervention may prevent unnecessary ED visits for withdrawal management in addition to complications of untreated OUD. Prior to the Bridge Clinic visit, cost of care was steadily increasing primarily related to unplanned care. Initial total cost rose in the 30 days after initial Bridge Clinic visit, but the trend in cost declined over a 180-day period with eventual lower total cost PMPM 180 days after the first Bridge Clinic appointment compared to the 30 days before. Given that many patients who would otherwise be unable to rapidly access treatment would not have engaged in care, the Bridge Clinic intervention may save payer outlays over time while also being a trigger for better care quality.

Bridge Clinics, including telemedicine, can improve performance in the cascade of OUD treatment quality outcomes, particularly seven day follow up after ED visit or hospital discharge, initiation of medication treatment within 14 days and at least 2 visits within the first 30 days of OUD diagnosis (Williams et al., 2018, Lin et al., 2023). Rapid engagement has been shown to improve 180-day retention, another recognized quality measure (Williams et al., 2023). The Bridge Clinic also provides a back-up for patients whose medication adherence is threatened by missed appointments, change in location or treatment provider, incarceration, or other barriers that could interrupt sustained treatment. Expansion of the Telemedicine Bridge Clinic model in coordination with existing community resources including OUD Centers of Excellence, EDs, urgent cares, and Federally Qualified Health Centers (FQHCs) offers a sustainable way to rapidly deliver care for complex, time-sensitive conditions including substance use disorders, associated medical illness such as HIV and viral hepatitis, and co-occurring mental health conditions. Building a seamless continuum of care with multiple convenient points of entry for patients has the potential to both improve outcomes and reduce costs. Further cost analyses done in partnership with payers will be important for informing development of novel payment approaches, including value-based arrangements, that would support capacity to scale services to areas where rapid access to MOUD treatment is limited.

## 5 Limitations

The observed cohort is small and included members of a single health plan. The sampling and unique nature of the program limited creation of an appropriately matched cohort, so we chose a pre/post evaluation that lessens causality inference but adds real world observations. The analysis may not account for all services provided to patients. Claims for Medicaid behavioral health benefits were not available as they are carved out from physical health benefits in Pennsylvania.

## 6 Conclusion

Utilization of a telemedicine bridge clinic was associated with buprenorphine initiation, linkage to ongoing care with retention including medication treatment, reduced unplanned care cost, and overall savings.

## Author Contributors

All authors have materially participated in the research and article preparation. All authors have approved the final article

## CRediT authorship contribution statement

**James Schuster:** Writing – review & editing, Supervision. **Donald M. Yealy:** Writing – review & editing, Supervision. **Justin Kanter:** Writing – review & editing, Writing – original draft. **Jessica Meyers:** Writing – review & editing, Writing – original draft, Conceptualization. **Dominic Vargas:** Writing – review & editing, Formal analysis, Data curation, Conceptualization. **Mary E. Winger:** Writing – review & editing, Formal analysis, Data curation, Conceptualization. **Michael J. Lynch:** Writing – review & editing, Writing – original draft, Formal analysis, Conceptualization.

## Declaration of Competing Interest

None
